# Redo-Posterior Neurectomy or Conservative Treatment for Recurrent Pain After Posterior Neurectomy in Anterior Cutaneous Nerve Entrapment Syndrome – A Case Comparison Analysis

**DOI:** 10.3389/jaws.2025.14828

**Published:** 2025-12-22

**Authors:** Tom ten Have, Marc R. M. Scheltinga, Elise Bekers, Willem A. R. Zwaans, Rudi M. H. Roumen

**Affiliations:** 1 Department of Surgery, Máxima Medical Centre, Veldhoven, Netherlands; 2 SolviMáx, Centre of Expertise for Complex Groin and Abdominal Wall Pathology, Máxima Medical Centre, Eindhoven, Netherlands; 3 NUTRIM School of Nutrition and Translational Research in Metabolism, Maastricht University, Maastricht, Netherlands; 4 Department of Pathology, Clinical Diagnostics PAMM, Eindhoven, Netherlands

**Keywords:** abdominal pain, chronic abdominal wall pain, acnes, neurectomy, surgery

## Abstract

**Objective:**

To provide insight into treatment outcomes of a redo-posterior neurectomy compared to conservative treatments in ACNES patients with recurrent pain after a previous successful posterior neurectomy.

**Summary Background Data:**

Most patients with chronic abdominal pain due to anterior cutaneous nerve entrapment syndrome (ACNES) benefit from a step-up treatment regimen including abdominal wall injections, pulsed radiofrequency, or surgery (an anterior or posterior neurectomy). However, some 20% of patients who underwent an initially successful posterior neurectomy develop recurrent pain. To date, studies regarding treatment options and outcomes of these patients are scarce.

**Methods:**

Eligible patients who received treatment in our center of expertise between January 2012 and February 2023 were analyzed using a questionnaire. Success was defined as a minimal 50% pain reduction for at least 3 months postoperatively using pain scores and Patient Global Impression of Change (PGIC).

**Results:**

Of 57 eligible patients, 37 (76% female, mean age 39 years) completed the questionnaire (65% response rate). Twenty had undergone a redo-posterior neurectomy whereas the remaining 17 patients continued conservative measures. Short-term surgical success rate was 95%. In the long-term (median 40 months), surgical treatment outcome was more successful compared to a conservative treatment regarding pain reduction (85% vs. 41%; *p* = 0.008) and PGIC (70% vs. 41%; *p* = 0.018).

**Conclusion:**

Based on the current study, redo-posterior neurectomy may be considered a beneficial option for ACNES patients with recurrent pain after an initially successful posterior neurectomy.

## Introduction

The anterior cutaneous nerve entrapment syndrome (ACNES) is a common cause of chronic abdominal wall pain [1, 2]. Although its management is challenging, studies from our center of expertise reported an almost 90% success rate using a strict treatment algorithm [3, 4]. For instance, approximately 45% of patients achieve sufficient pain relief following minimally invasive tender point injections or pulsed radiofrequency (PRF) treatment [3–7]. Unresponsive individuals are usually counselled for a neurectomy of end branches of the intercostal nerves in the anterior abdominal wall [2, 6, 8]. Two surgical options are included in the treatment algorithm. At first, an anterior neurectomy is proposed. If unsuccessful, a posterior neurectomy is suggested. Long-term success rates are 60% and 50%, respectively [3, 9, 10]. However, outcome after the posterior neurectomy is dependent on earlier surgical success [3, 10]. If the anterior neurectomy was initially (>3 months) beneficial, long-term success rate of the posterior neurectomy is 60%. In contrast, a 40% success rate is attained after an previously unsuccessful anterior neurectomy [10].

The recurrence rate after a successful posterior neurectomy approximates 20% [10]. Consensus on treatment options in these recurrent pain patients is lacking. As a posterior neurectomy is considered a final surgical option, these patients often continue to undergo a variety of conservative (mainly pharmacotherapeutic) treatment options. Interestingly, the recent cohort study by our center reported a remarkable high success rate after a repeated retrorectus exploration termed a redo-posterior neurectomy [10].

Aim of the present study is to compare outcomes of a redo-posterior neurectomy with conservative treatments in patients experiencing recurrent pain after a previous successful posterior neurectomy. Recurrent pain was defined as the return of pain after a significant pain reduction (>50%) for at least 3 months. Operative findings of the redo-posterior neurectomy will also be discussed. Based on clinical experience at our expertise center with both surgical and non-surgical treatments in ACNES patients, it was hypothesized that patients with recurrent pain after a previous successful posterior neurectomy will have greater benefit from redo-posterior neurectomy compared to non-surgical treatments.

## Materials and Methods

Patient eligibility was based on a cross-sectional cohort study on long-term treatment outcomes of the posterior neurectomy for ACNES patients who were operated between January 2012 and January 2022 in SolviMáx, an acknowledged center of expertise for complex groin and abdominal wall pathology in the Máxima Medical Center [10]. In this recently published study, all patients who underwent an unilateral posterior neurectomy following our standard treatment protocol during this specific time period were included. Exclusion criteria included inguinal nerve involvement, missing follow-up data, inability to read Dutch, or when deceased. A total of 379 included patients were sent a questionnaire [10].

For the present study, only patients with recurrent pain after a previously successful posterior neurectomy were eligible. A successful outcome was defined as a minimal 50% pain reduction for at least 3 months after the posterior neurectomy [11]. If the pain recurred after this period, it was classified as recurrent pain. Only patients who completed a questionnaire regarding previously received treatment(s) and respective outcomes, as well as their present (long-term) treatment outcome based on pain relief (using the Numeric Rating Scale NRS; range 0–10) and patient global impression of change (PGIC) were studied [12]. No new data were obtained. The Daily Board of the Medical Ethics Committee (METC) of the Máxima Medical Center confirmed that no further ethical approval was required (METC number: N20.065) [10].

### Outcome Measures

Baseline data extracted from electronic patient files included demographics (age, body mass index, gender) and pain characteristics as assessed during the first outpatient visit (mean NRS before treatment, duration of pain before referral and chronic abdominal wall pain score [13]). Short-term treatment outcomes of the redo-posterior neurectomy as scored during the four to six weeks postoperative follow-up consult were collected.

Primary outcomes were short- and long-term treatment success after redo-neurectomy, long-term treatment success after conservative treatment, and long-term treatment satisfaction based on PGIC outcomes. Treatment success was defined according to IMMPACT (Initiative on Methods, Measurement, and Pain Assessment in Clinical Trials) recommendations as a minimal 50% pain reduction or a minimal four points pain reduction using NRS [11]. PGIC scores of “very much improvement” or “much improvement” were considered clinically significant improvements [11].

Secondary outcomes included perioperative findings including macroscopic identified neuroma, complication rates, time till recurrence, and recurrence rates.

### Redo-Posterior Neurectomy

A redo-posterior neurectomy is essentially a re-exploration of the abdominal wall in the retrorectus plane aiming for a repeated posterior neurectomy via the previous scar [3, 10]. The anterior rectus sheath is exposed and potential (newly sprouting) perforating neurovascular bundles are coagulated. The anterior rectus sheath is reopened via a longitudinal or oblique incision and the rectus abdominis muscle is retracted medially. Any lateral or medial neurovascular bundles in the retrorectus space are identified and coagulated or ligated. The retrorectus plane is explored 5 cm above and below the tender point to identify possible neuromas, specifically at the lateral border. The wound is closed in layers following infiltration with 20 mL of a long acting local anesthetic agent.

### Data Analysis

Data were tested for skewness. Normally distributed data were presented as means with 95% confidence intervals, while skewed and categorical data were presented as median with interquartile ranges (Q1-Q3). Normally distributed continuous data were analyzed using the Student’s t-test, skewed and categorical data with the Mann-Whitney *U* test, and binary data with the Fisher’s exact test. A multivariable logistic regression analysis was performed to assess whether the time until recurrence and treatment type were associated with treatment success, presented as odds ratio (OR) with 95% confidence intervals. SPSS software Version 22.0 for Windows (IBM, Armonk, NY, USA) was used for the analyses. A *p*-value of <0.05 was considered statistically significant.

## Results

A total of 57 patients fulfilled study criteria, and 37 completed the questionnaire (65% response rate). Baseline characteristics are presented in Table 1. Short-term treatment success of a redo-posterior neurectomy were 95% (n = 19/20) among responders and 83% (n = 5/6) among non-responders.

**TABLE 1:** Characteristics of patients with recurrent pain after posterior neurectomy (n = 57).

Characteristics	Responders (n = 37)	Non-responders (n = 20)
Age (years)	39 (34–44)	35 (30–41)
Sex ratio, M:F	1:3.1	1:4
BMI (kg/m^2^)^a^	26 (23–30)	25 (22–28)
NRS before treatment^a^	7 (7–8)	8 (7–9)
Duration of pain (months)^a^	24 (10–49)	17 (6–35)
Abdominal wall score^a^	14 (12–15)	14 (13–16)
Received redo-posterior neurectomy	20 (54%)	6 (30%)

Presented as means with 95% confidence interval or absolute count with percentages.

BMI, Body Mass Index; NRS, Numeric Rating Scale.

^a^ Median with interquartile range (Q1-Q3).

### Intraoperative Findings

**FIGURE 1:**
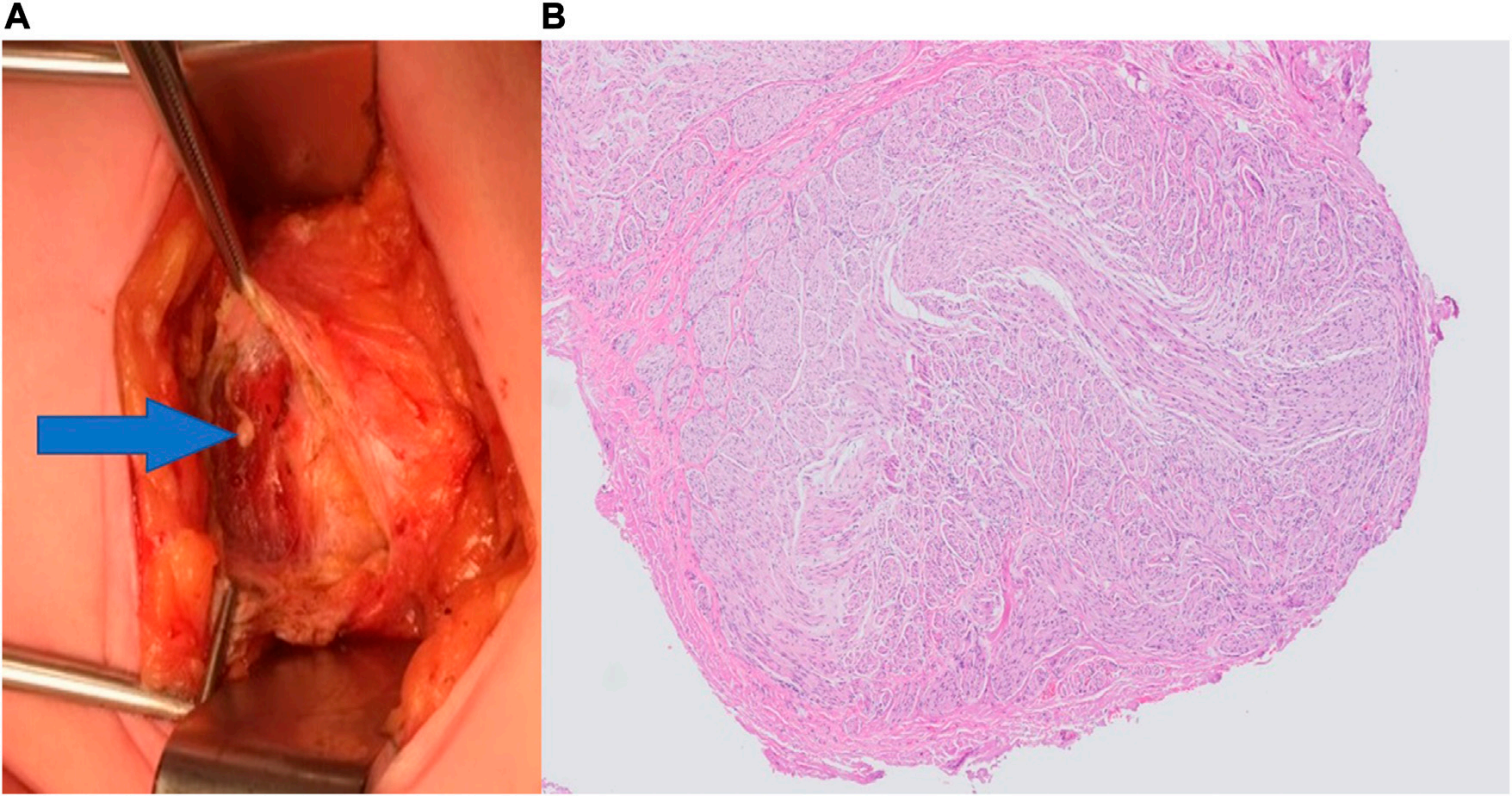
**(A)** Macroscopic photograph of a neuroma (arrow) located beneath the anterior rectus sheath, as seen during Redo-posterior neurectomy. **(B)** Hematoxylin and eosin staining (H&E) at ×50 magnification showing an overview of a neuroma.

Twenty of the 37 study patients (54%) underwent a redo-posterior neurectomy. Median time until recurrence after the posterior neurectomy was 35 months (range 3–100 months). Nerve structures were identified at the level of both the anterior and posterior rectus fascia in all 20 patients. Nerve tissue were confirmed in all cases where pathological assessment was performed (n = 11). The pathologist diagnosed a neuroma (Figure 1) in three cases (15%). Short-term success rate was 95% (n = 19/20). One patient reported a wound abscess necessitating partial wound opening.

Long-term treatment success was 70% (n = 14/20; median follow-up of 40 months, range 7–111 months). The median NRS decreased from 7 before treatment to 1 at the end of follow-up (Table 2A). Five of the initially successful redo-posterior neurectomy patients reported recurrent pain after 9, 10, 12, 16, and 72 months, respectively. One patient was successfully treated with a single transverse abdominis plane (TAP) block, while the remaining four patients underwent a redo-neurectomy (fourth neurectomy), all of which were successful (in the short-term). Again, nerve structures were identified in all four cases. One patient reported a recurrence 3 years after the fourth neurectomy, for which a successful redo-neurectomy (fifth neurectomy) was performed, revealing a neuroma.

**TABLE 2:** Pain scores using the numeric rating scale before and after treatment for A. Surgical neurectomy group and B. Non-surgical treatment group.

Treatment outcome	NRS before start treatment	NRS at the end of follow-up
A. Surgical neurectomy treatment outcome		
Succes (n = 17)	7 (7–8)	1 (0–5)
Failure (n = 3)	9 (8–9)	8 (8–9)
B. Non-surgical treatment outcome		
Succes (n = 7)	7 (7–9)	3 (1–5)
Failure (n = 10)	7 (6–8)	7 (6–8)

Presented as median with interquartile range (Q1-Q3).

NRS, Numeric Rating Scale.

### Findings of Ongoing Conservative Treatment

Seventeen of the 37 patients (46%) received non-surgical pain treatments. Median time between recurrence of pain and the posterior neurectomy was 5 months (range 3–49 months), significantly shorter compared to the patients who received a redo-posterior neurectomy (*p* = 0.007). Non-surgical treatments included pharmacotherapy, manual therapy, local anesthetic injections and/or pulsed radiofrequency treatment. Short-term treatment outcomes were unknown as these treatments were often not performed in our center.

Long-term treatment success at 40 months was 41% (n = 7/17), significantly lower than the surgical treatment group (*p* = 0.008). In this group the median NRS scores decreased from 7 to 3 (Table 2B). There was no single treatment modality that distinguished between treatment successes and failures, all patients received different combinations of treatments.

### Long-Term Patient Satisfaction

**FIGURE 2:**
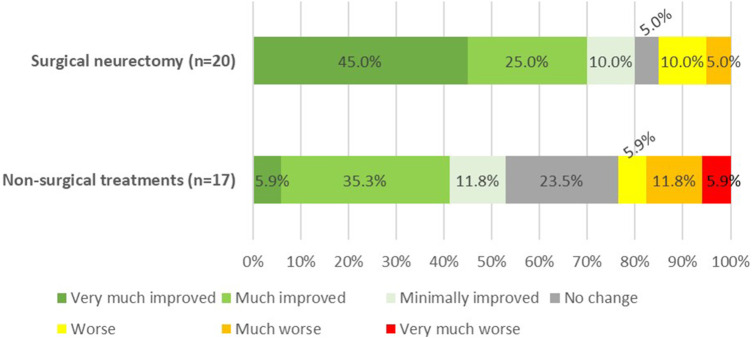
Patients global impression of change (PGIC) at 40 months follow-up after the redo-posterior neurectomy (*p* = 0.018).

Based on the PGIC scores, 70% of the surgical group reported a significant improvement. Although this percentage is slightly lower than the treatment success based on NRS, it remained significantly higher compared to the 41% in the non-surgical group (*p* = 0.018) (Figure 2). None of the patients experienced bulging of the rectus abdominis muscle during follow-up.

### Treatment Type vs. Time Until Recurrence

The multivariate logistic regression analysis of all included patients demonstrated that treatment type (redo-posterior neurectomy) was associated with an odds ratio for treatment success of 3.51 (95% CI 0.70–17.6), although not statistically significant (*p* = 0.160). A longer time to recurrence was significantly associated with an odds ratio of 1.08 (95% CI 1.00–1.16, *p* = 0.049).

## Discussion

This study is the first to describe the option and outcome of a redo-posterior neurectomy in ACNES patients with recurrent pain after a previous successful posterior neurectomy. Although the proposed re-exploration in the retrorectus plane should be considered as a repeat posterior neurectomy, the success rate in this small cohort is remarkably high. In addition, patients who underwent surgery also had a significantly better outcome based on both NRS and PGIC compared to patients who received non-surgical treatments.

The observed 95% short-term and 70% long-term success rates are higher compared to both an anterior neurectomy (70% and 60%) and a posterior neurectomy (60% and 50%) [6, 9, 10]. This finding is in line with the reported tendency of higher success rates after a previously successful neurectomy [10]. This may seem theoretically plausible as the pain has been demonstrated to be responsive to earlier peripheral surgical treatment. Nerve structures were found during all redo-posterior (and subsequent) neurectomies, suggesting nerve regeneration as a possible explanation for the recurrent pain. This may also explain why repeating the same surgical procedure (up to a fifth redo-neurectomy in this study) can still be successful in cases of recurrent pain. It was earlier reported that nerve injury may change a neuron state from primary signaling to a regenerating function leading to sprouting of the proximal nerve. These nerve sprouts can possibly re-innervate rectus abdominis muscle tissue, or may result in a neuroma if there is no guidance structure for the proximal nerve ending [14, 15]. The present series just identified three neuromas (15%) which is quite similar to a reported 20% neuroma incidence in another ACNES population [9]. Although neuroma formation after neurectomy is a feared phenomenon [16], it seldom appears the cause of clinically relevant pain. It may well be that re-innervation of a previous painful area (muscle/skin) is a more prominent cause of recurrent pain.

Some studies advice to perform a laparoscopic proximal neurectomy in the transverse abdominal plane for recurrent or refractory ACNES [17, 18]. Although this approach is feasible, success rates are unknown. Moreover, intercostal nerves have a motoric function of the oblique and rectus abdominal wall muscles as well. For instance, 1% of patients reported bulging of the abdominal wall after a posterior neurectomy, likely related to rectus abdominis muscle denervation [10]. A more proximal neurectomy will increase the risk of rectus and lateral abdominal wall muscles bulging as sometimes observed after thoracic or open retroperitoneal surgery [19, 20]. Therefore, a redo-neurectomy as presented in this paper may be preferable in ACNES patients with recurrent pain. Interestingly, a TAP block with a local anesthetic agent is a possible less invasive treatment option in this population and may also be used as a diagnostic tool. This approach resulted in a long term pain free status in one of our patients presented in this series. We are currently engaged in studying the role of a TAP block in this specific patient population.

Almost half of our patients (n = 17) received non-surgical treatments by pain specialists who were not related to our institution. Therefore, short-term success rates of these different therapies are unknown. In the long term, success based on NRS (85% vs. 41%) and patient satisfaction based on PGIC (70% vs. 41%) were both significantly less favorable in these patients. However, a degree of selection bias may be present. As demonstrated by the logistic regression analysis, a longer time until recurrence was a significant predictor of treatment success (OR 1.08). The time until recurrence was significant longer in the surgery group (35 months vs. 5 months). Moreover, the treatments were not controlled, resulting in a variety of approaches due to the lack of consensus on the optimal treatment option. Consequently, the decision on a certain treatment was based on the expert opinion of the treating physician and patient preference. It is conceivable that both surgeon and patient were less inclined to opt for a redo-neurectomy when pain recurred a few months after the posterior neurectomy, and leading instead to the selection of conservative treatments. As a result, it remains uncertain whether these patients might have benefitted from a redo-neurectomy. Notably, some patients with recurrence pain only a few months after posterior neurectomy experienced a successful outcome after redo-neurectomy. In contrast, the logistic regression analysis showed a trend favoring the redo-neurectomy group (OR 3.51), although not reaching statistical significance, which is likely due to the small sample size. Despite the lack of statistical significance, the observed odds ratio and trend may suggest a clinically relevant benefit of redo-neurectomy, warranting further (prospective) investigation in larger cohorts.

Although the surgical techniques for posterior neurectomy and redo-neurectomy remained consistent over the years, it is possible that early in the inclusion period, surgeons were more hesitant to perform redo-neurectomy due to limited familiarity with the procedure. Over time, they gained experience, potentially leading to a lower threshold for recommending redo-neurectomy.

**FIGURE 3:**
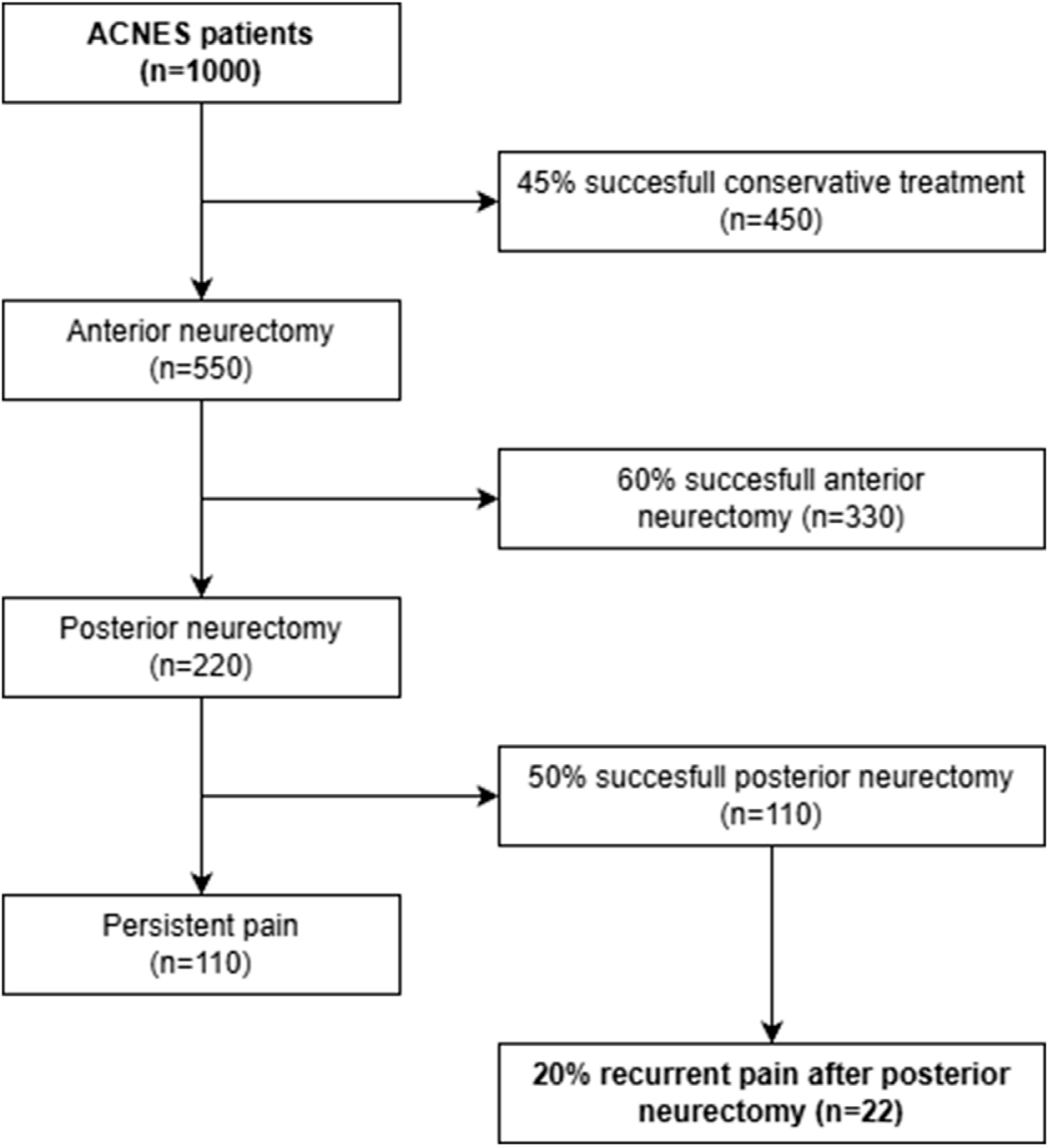
Flowchart illustrating the number of ACNES patients who will experience a recurrent pain after posterior neurectomy, based on reported long-term treatment success in previous studies [3–6, 9, 10].

Additional limitations of the present survey study include potential non-response bias. Despite an acceptable response rate of 65%, patients who did not benefit from treatment may be less likely to participate in such surveys. Although as a center of expertise, we often serve as a last resort option for patients living with this type of chronic abdominal wall pain. These patients generally are motivated to participate in research, hoping that it may lead to new treatment options in the future. Although the current study included just 37 patients, their baseline characteristics are comparable to the entire cohort of 359 patients who received the questionnaire in the initial long-term follow-up study of the posterior neurectomy [10], suggesting that this subgroup is representative of the ACNES population. In addition, based on the current knowledge and data, only 2% of ACNES patients treated by our complete algorithm will develop recurrent pain after a posterior neurectomy (Figure 3) [3–6, 9, 10].

In conclusion, this case comparison study demonstrates a trend favoring redo-posterior neurectomy in ACNES patients with recurrent pain at least 3 months after an initially successful posterior neurectomy. However, prospective studies, ideally a randomized controlled trial, is necessary to determine whether redo-posterior neurectomy represents the optimal treatment option for this small group of ACNES patients.

## Data Availability

The raw data supporting the conclusions of this article will be made available by the authors, without undue reservation.
